# Photodynamic therapy with chlorins for diffuse malignant mesothelioma: initial clinical results.

**DOI:** 10.1038/bjc.1991.474

**Published:** 1991-12

**Authors:** H. B. Ris, H. J. Altermatt, R. Inderbitzi, R. Hess, B. Nachbur, J. C. Stewart, Q. Wang, C. K. Lim, R. Bonnett, M. C. Berenbaum

**Affiliations:** Department of Thoracic and Cardiovascular Surgery, University of Bern, Switzerland.

## Abstract

**Images:**


Br. J. Cancer (1991), 64, 1116–1120 © Macmillan Press Ltd., 1991

### Photodynamic therapy with chlorins for diffuse malignant mesothelioma: initial clinical results

H.-B. Ris[1], H.J. Altermatt[2], R. Inderbitzi[1], R. Hess[1], B. Nachbur[1], J.C.M. Stewart[3], Q. Wang[4], C.K. Lim[4], R. Bonnett[5], M.C. Berenbaum[6] & U. Althaus[1]

*Departments of [1]Thoracic and Cardiovascular Surgery and [2]Pathology, University of Bern, Switzerland; [3]Scotia Pharmaceuticals Ltd, Guildford; [4]Medical Research Council Laboratories, Surrey; [5]Department of Chemistry, University of London; and [6]Department of Experimental Pathology, St Mary's Hospital Medical School, London, UK.*

**Summary** Four patients underwent intraoperative photodynamic therapy after surgery with meso-tetra-(hydroxyphenyl)-chlorin (mTHPC-PDT) for diffuse malignant mesothelioma. Preliminary procedures were performed in two patients in order to establish the efficacy of mTHPC-PDT and to optimise its tumoricidal effect. The tumoricidal effect was related to the mTHPC dose, light dose and the time interval between sensitation and activation. 0.3 mg $kg^{-1}$ mTHPC activated after 48 h with 10 Joules $cm^{-2}$ of non-thermal laser light at 650 nm resulted in a 10 mm deep tumour infarction, due to tumour vessel necrosis and thrombosis. The mTHPC tissue concentration was up to 14 times higher in the tumour than in normal tissues. Skin photosensitivity was mild, dose dependent and occurred 3 to 10 days after administration of mTHPC. According to the results obtained, intraoperative mTHPC-PDT was performed following pleuropneumonectomy in two, pleurectomy and lobectomy in one and pleurectomy in one patient. Ten Joules $cm^{-2}$ were delivered to the diaphragm and the costophrenic sulcus and 5 Joules $cm^{-2}$ to the remaining thoracic cavity. The postoperative course was marked by loss of appetite, fluid retention, hypoproteinemia and severe chest pain. One patient succumbed from aspiration pneumonia. The remaining patients developed no neural or vascular alterations and no bronchial stump insufficiency during follow-up. mTHPC-PDT following surgical tumour resection deserves further evaluation in good risk patients with diffuse malignant mesothelioma.

Diffuse malignant mesothelioma spreads on pleural and peritoneal surfaces with invasion of the underlying structures, first of all of the diaphragm. There is no cure at present. Patients succumb in general from relentless local progression of the disease regardless of the treatment performed and not from distant metastatic spread, indicating that local control is not effective even if extended resections have been performed (Faber, 1988). Improved local control does require additional measures, but the disease responds poorly to radio- and chemotherapy (Lerner *et al.*, 1983). As photodynamic therapy (PDT) has been reported to be effective in human mesothelioma xenografts (Feins *et al.*, 1990), it might allow for an appropriate 'clean-up' of the thoracic cavity after surgery. For clinical purposes, the currently used sensitisers for PDT are haematoporphyrin derivatives (HpD) and dihaematoporphyrin ether (DHE) (Dougherty *et al.*, 1990). However, PDT with meso-tetra-(hydroxyphenyl)-chlorin (mTHPC) was superior to DHE-PDT with respect to antitumour activity and tissue selectivity in rodents without causing significant toxicity (Berenbaum, 1989). mTHPC might therefore be better fitted to large surface PDT as required for diffuse malignant mesothelioma treatment. A pilot study was done to evaluate mTHPC-PDT for diffuse malignant mesothelioma with respect to its antitumour activity and the feasibility of a combined modality approach under clinical conditions.

### Patients and methods

Four patients underwent mTHPC-PDT for diffuse malignant mesothelioma. Each patient was informed in detail about the experimental nature of the procedure and consent was obtained from each patient and from the local Human Investigations Committee of our institution.

The four men were aged 46 (patient 1), 48 (patient 2), 65 (patient 3) and 50 years (patient 4), all having had possible occupation related exposure to asbestos. The main symptoms at admission were dyspnoea due to pleural effusion, chest pain and loss of weight. There was no evidence of disease in the peritoneal and contralateral chest cavity on CT-scans at admission. The right side was involved in patients 1, 2 and 4 and the left in patient 3. Previous biopsies revealed an epithelial (Figure 1a), a biphasic (Figure 2a), a sarcomatous and a mixed type of mesothelioma in the four patients and was confirmed in every case by immunohistologic examinations.

### *Preliminary PDT*

To establish the efficacy of mTHPC-PDT and to optimise its tumouricidal effect, preliminary PDT was performed in patients 1 and 2 prior to its definitive application. Modulations of mTHPC dose, light dose and of the time interval between mTHPC application and activation were tested.

mTHPC (Scotia Pharmaceuticals Ltd, Guildford, UK) was dissolved in 20% ethanol, 30% polyethylene glycol 400 and 50% $H_2O$ and administered over 15 min i.v. through a bacterial filter under sterile conditions within 60 min of preparation. Argon-pumped dye laser light of 650 nm (Coherent Innova 200 and Dye CR 599, GMP SA, Lausanne, Switzerland) was delivered through a sterilised optical fibre on tumour areas of 3 cm diameter. The power at the end of the optical fibre was measured with a power meter, allowing for a power density of 0.1 Watt $cm^{-2}$ on the treated surfaces (non-thermal surface irradiation). No protection of the skin from the operating theatre lights was performed. Normal light sources were used for laryngoscopy and bronchoscopy during double lumen intubation which was performed for each procedure. Five days after the light delivery the areas were biopsied and the histologic specimen compared to those of the untreated areas. Light delivery and biopsy procurement were performed on both patients twice by thoracoscopy and once through a small thoracotomy to obtain exact irradiance geometry and a cross sectional profile of the PDT related tumour destruction. The time interval between the three PDT was 3 weeks. mTHPC plasma concentrations were measured at regular intervals by high performance liquid chromatography up to 9 days after administration. Patients were cautioned to avoid direct sunlight for about 2 weeks but were encouraged to test skin sensitivity by daily exposure of the hand for a short period of time.

Correspondence: H.-B. Ris, Klinik für Thorax- Herz- und Gefässchirurgie, Inselspital, 3010 Bern, Switzerland.
Received 7 June 1991; and in revised form 13 August 1991.

**Figure 1** Photodynamic therapy with mTHPC for diffuse malignant mesothelioma: **a**, Morphology of the untreated tumour of patient 1; **b**, Tumour infarction due to tumour vessel necrosis and thrombosis after 0.075 mg $kg^{-1}$ mTHPC and 10 Joules $cm^{-2}$, time interval 24 h; **c**, 10 mm deep tumour necrosis in the centre and the periphery of the treated area after 0.3 mg $kg^{-1}$ mTHPC and 10 Joules $cm^{-2}$, time interval 48 h. The specimens for histology were taken 5 days after PDT (Haematoxylin-Eosin, bar = 200 $\mu$m).

**Figure 2** Photodynamic therapy with mTHPC for diffuse malignant mesothelioma: **a**, Morphology of the untreated tumour of patient 2; **b**, Balloonated basophilic tumour cells of questionable viability and prominent interstitial edema after 0.15 mg mTHPC $kg^{-1}$ and 2 Joules $cm^{-2}$, time interval 24 h. The specimens for histology were taken 5 days after PDT. (Haematoxylin-Eosin, bar = 200 $\mu$m).

*Intraoperative PDT following surgery*

According to the results obtained from preliminary PDT, intraoperative PDT following surgical tumour resection was performed with 0.3 mg $kg^{-1}$ mTHPC administered 48 h prior to light delivery in all four patients. The surgical procedure consisted of an extrapleural pneumonectomy in patients 1 and 2, a pleurectomy in patient 3 and a pleurectomy with resection of the lower lobe in patient 4. A cleavage plain between normal tissue and tumour was found in patients 1 and 2 at the chest wall and the mediastinum, and in patient 4 at the chest wall, the mediastinum and the upper lobe. No reasonable plain was found at the chest wall, the mediastinum and the lung in patient 3 with a sarcomatous type of tumour. The diaphragm was debulked yet preserved in all four patients in order to keep intact this natural barrier to the peritoneal cavity. The pericardium was removed in patient 1 and 2, preserved in patient 3 and partially removed and replaced by a vicryl mesh in patient 4. After the resection, the light was delivered through the open chest wound, with a dose of 10 J $cm^{-2}$ to the diaphragm and the costophrenic sulcus and of 5 J $cm^{-2}$ to the remaining cavity (including the lung in patients 3 and 4). The diameters of the light spots varied from 5 to 12 cm, according to the geometry of the area treated. To reduce additional loss of energy and divergence of the laser beam, the light was delivered through a bare fibre rather than a lens. The spots were therefore overlapped to prevent unequal light distribution by the bare fibre. The maximal laser power output at the end of the fibre was 1.5 Watt in patient 1, 2 and 3. Patient 4 was treated by a new high power laser system, allowing for 4 Watt at the end of the fibre at 650 nm. The heart was shielded from direct irradiance by a moist towel in patients 1 and 2. mTHPC concentrations were measured in the tumour and in normal tissues 48 h after mTHPC-administration.

### Results

*Preliminary PDT*

*Tumour response to mTHPC-PCT* 0.075 mg $kg^{-1}$ mTHPC and 10 Joules $cm^{-2}$ delivered after 24 h resulted in frank necrosis of approximately 50% of tumour on semi-serial sections investigated from treated areas (diameter of biopsy specimen: 10 mm). The histologic pattern of necrosis was consistent with tumour infarction. Tumour vessels showed a fibrinoid necrosis of the vessel wall and thrombosis. The tumour necrosis extended from the vascular watershed towards the vessels whereas the remaining 50% of tissue around the vessels appeared edematous and possibly viable (perivascular sparing, Figure 1b). Specimens taken from untreated tumour areas served as controls and showed no spontaneous tumour necrosis (Figure 1a).

0.15 mg mTHPC $kg^{-1}$ activated after 24 h with 10 Joules $cm^{-2}$ resulted in a 50% to 80% infarction related tumour destruction. However, balloonated basophilic tumour cells of questionable viability and prominent interstitial edema were observed with 0.15 mg mTHPC $kg^{-1}$ and 2 Joules $cm^{-2}$ delivered after 24 h (Figure 2b). No spontaneous tumour necrosis was found in biopsy specimens from adjacent but untreated tumour areas (Figure 2a).

0.3 mg mTHPC $kg^{-1}$ and 10 Joules $cm^{-2}$ delivered after 24 h resulted in a 10 mm deep complete tumour necrosis in the centre but with preserved cells in the periphery of the treated areas. No viable tumour cells were observed in the periphery when the same light dose was applied after 48 h (Figure 1c). Higher light doses (up to 40 Joules $cm^{-2}$) did not lead to deeper tumour destruction than that observed with 10 Joules $cm^{-2}$.

*Skin photosensitivity* The only side effect observed after mTHPC administration was a mild, dose dependent skin photosensitivity. It appeared 3 to 10 days after administration of mTHPC and lasted for 2 to 3 days. Photosensitivity was observed after direct and indirect (closed window) exposure to sunlight, but not under normal room light conditions. No adverse effect was observed from the operating theatre lights nor from the lights used for laryngoscopy and bronchoscopy during double lumen intubation, although these lights contain a high percentage of red light.

*mTHPC plasma, urine and tissue concentrations* Plasma concentrations followed a first order kinetics after i.v. application and the half-life time was 12 h. An average plasma concentration of $14.6 \pm 7.6\ \mu g\ 100\ ml^{-1}$ was measured 48 h after administration of 0.3 mg mTHPC $kg^{-1}$. At this time, the mTHPC concentration in the tumour was $1.4\ \mu g\ g^{-1}$, in the bronchus, pulmonary artery and pulmonary vein wall of the resected specimen $0.2\ \mu g\ g^{-1}$, $0.3\ \mu g\ g^{-1}$ and $0.1\ \mu g\ g^{-1}$ and in muscle and skin tissue $0.2\ \mu g\ g^{-1}$ and $0.1\ \mu g\ g^{-1}$ respectively.

Nine days after administration, mTHPC was still detectable in the plasma ($4\ \mu g\ 100\ ml^{-1}$). No mTHPC or metabolites were identified in urine samples at any time.

### *Intraoperative PDT following surgery*

An additional 2 h were required for PDT of the thoracic cavity in patients 1, 2 and 3 after completion of the surgical tumour resection. Appropriate exposure of the costophrenic sulcus was obtained by manual retraction of the diaphragm. Difficulties were encountered in ensuring equal and sufficient light distribution to the anterior chest wall in these three patients and to the entire surface of the collapsed lung in patient 3. Patient 4 was treated with a new high power laser system and the overall PDT treatment time was reduced to 50 min. Appropriate light delivery to the costophrenic sulcus, the anterior chest wall and to the remnant lung was much easier in this patient due to the markedly reduced treatment time.

*Postoperative course* Loss of appetite, fluid retention (up to 8 kg), hypoproteinemia ($50.4 \pm 7\ g\ l^{-1}$) and more severe chest pain than anticipated from the surgical procedure *per se* were specific side effects following this combined approach. Serum creatinine values and urine output were normal throughout. SGOT, SGPT and alkaline phosphatase values were slightly increased in the first postoperative days, but did not exceed 3-fold of the normal range. CK values were not higher than after surgery *per se* ($1,000\ U\ l^{-1}$) in patients 1, 2 and 3; however, they were increased in patient 4 ($3,800\ U\ l^{-1}$). The CK-MB fraction was not increased in any patient. The chest pain was controlled by opiods or peridural analgesia and disappeared after about 1 week. Retained fluids were spontaneously mobilised 3 to 5 days after the operation. The clinical course in patient 4 did not differ from that in patients 1 and 2, despite the higher power density applied in this patient.

Patients 1, 2 and 4 were discharged after 3 weeks, but patient 3 succumbed from a massive aspiration induced pneumonia of his contralateral lung on the sixth postoperative day. The autopsy revealed PDT induced necrosis of the remnant tumour throughout the chest cavity and the lung surface. The depth of necrosis, however, varied with the area treated, and ranged from 0.5 to 1 cm at the diaphragm (Figure 3a) and the costophrenic sulcus and from 0.3 to 0.5 cm at the remaining sites of the cavity, including the lung surface. Even in the presence of full thickness necrosis of the adjacent tumour, underlying structures such as the aorta, nerve ganglion cells and the oesophagus were spared (Figure 3b and 3c). However, chondrocytes and osteocytes of the ribs and smooth muscle cells of the aorta adjacent to the destroyed tumour were also altered, due to PDT induced damage of nutritive vessels (Figure 3d). The lung showed small foci of subpleural alveolitis under the destroyed tumour.

*Follow-up* No insufficiency of the bronchial stump and no vascular alterations were observed in the remaining three patients during follow-up. Patient 2 showed a diffuse weakness of his right arm without evidence of a lesion of the peripheral nerves, however. CT-scans revealed no structural alterations of the mediastinal organs 3 months after the operation. There was no evidence of disease in patients 2 and 4 on CT-scans at the time, however, patient 1 showed contralateral disease and anterior chest wall invasion at a previous biopsy site.

### Discussion

The goal of PDT is selective tumour eradication whilst sparing adjacent normal tissue. A sensitiser with preferential uptake by tumour tissue is administered and activated by a non-thermal dose of laser light of a specific wavelength, leading to free radical formation and destruction of the target tissue. Intraoperative PDT of a tumour bed following surgery is a promising concept for tumours not removable with the required margins of tumour-free tissue. Pilot studies have been done in this respect with HpD and DHE for retroperitoneal sarcomas (Nambisan *et al.*, 1987), residual or recurrent colorectal cancer in the pelvis (Herrera-Ornelas *et al.*, 1985), diffuse malignant mesothelioma (Pass *et al.*, 1990; Lofgren *et al.*, 1991) and for disseminated intraperitoneal malignancies (Sindelar *et al.*, 1991). The results of these studies indicate that large surface PDT is feasible: however, the tumouricidal effect for intraoperative large surface PDT has not been proven in these reports. Moreover, the delivered light dose ranged from 3 to 400 Joules $cm^{-2}$ and the optimal dose still needs to be clarified. A major hindrance in treating large surfaces is their irregular geometry which renders uniform light delivery difficult. Efficacy and tumour selectivity of PDT are crucial for this purpose. Both depend on the sensitiser used and, according to the given sensitiser, on the interplay of its dose, the light dose and the time interval between application and activation. To overcome the shortcomings of HpD and DHE, new sensitisers have been developed with improved properties in this respect (Pandey *et al.*, 1991). Among chlorins, mTHPC has shown excellent tumour eradication and tissue selectivity in rodents, requiring only 10 Joules $cm^{-2}$ to induce tumour necrosis of 0.6 cm depth, and without apparent side effects and minimal skin sensitivity (Berenbaum *et al.*, 1989). Furthermore, mTHPC strongly absorbs at 650 nm. This wavelength penetrates tissue better than that required for HpD or DHE activation. The

**Figure 3** Combined modality approach for diffuse malignant mesothelioma. Autopsy findings in patient 3, 6 days after PDT: **a** 5 to 10 mm deep necrosis of the tumour invading the diaphragm (bar = 2 mm); **b**, destroyed tumour with preservation of an underlying nerve ganglion (*fixation related artifact, bar = 200 μm); **c**, destroyed tumour invaded the aorta with preservation of the vessel wall (bar = 2 mm); **d**, altered smooth muscle cells of the aorta adjacent to destroyed invading tumour (bar = 200 μm) (Haematoxylin-Eosin).

effective penetration depth steeply increase in soft tissues between 600 and 650 nm (Wilson & Patterson, 1990). Our initial clinical results demonstrate a preferential uptake of mTHPC in tumour tissue. The mTHPC concentration was up to 14 times higher in the tumour than in the skin and other normal tissues. In contrast, for HpD and DHE a 2 to 4 ratio of tumour to skin concentration was reported (Gomer & Dougherty, 1979; Moan *et al.*, 1987). Furthermore, a light dose of 10 Joules $cm^{-2}$ caused a 10 mm deep tumour necrosis after administration of 0.3 mg $kg^{-1}$ mTHPC to our patients. This efficacy has not been reported for DHE- or HpD-PDT. In addition, the only side effect caused by this mTHPC dose was a mild skin sensitivity of shorter duration than observed after HpD or DHE application. Patients have to avoid outdoor sunlight for about 10 days after administration of mTHPC, as opposed to at least 1 month after HpD and DHE application.

As expected from previous results (Nelson *et al.*, 1990), tumour vessels seem to be the primary target for mTHPC-PDT, leading to necrosis of the vessel wall, thrombosis and subsequent tumour infarction (Figure 1b). However, the different morphology obtained after low light doses (Figure 2b) suggests a different mechanism of action and may be related to direct cell alteration by PDT.

Our results indicate that the tumouricidal effect depends on mTHPC dose, light dose and the time interval between sensitation and activation. Small mTHPC and light doses and a shorter time interval resulted in decreased tumour destruction. Variations of mTHPC short dose had a greater impact in this respect than variations of light dose. However, the optimal configuration has yet to be defined. It was noteworthy that the autopsy findings showed no adverse altering of the PDT-induced tumouricidal effect by previously performed debulking surgery.

Although exposed to the same light dose as the remnant tumour, the apparent absence of extensive muscle necrosis and of damage to the bronchial, vascular and neural structures suggest some reasonable degree of treatment selectivity for mTHPC-PDT under clinical conditions. However, the large surface treatment caused substantial additional burden to the patients in the postoperative course. The autopsy findings also raised concern about what degree normal tissue can be spared in the case of invading tumours. Although normal tissue damage was restricted to a few cell layers bordering the destroyed tumour, these results indicate further attention should be paid towards late sequelae of the involved underlying structures. Decreasing the mTHPC dose while increasing the light dose might improve tissue selectivity, as has been reported of DHE-PDT (Dougherty *et al.*, 1990).

Sufficient and uniform light delivery to all areas of the large and complex shaped thoracic cavity without exceeding a reasonable overall treatment time is mandatory for PDT of diffuse malignant mesothelioma. A high power laser system is now available, allowing for 4 Watt power output at the end of the treating fibre at 650 nm, with Kiton Red as dye. As shown in patient 4, this reduces the overall treatment time required for PDT to less than 1 h and does not increase morbidity despite the higher power density applied. Sophisticated light delivery devices (DeLaney *et al.*, submitted for publication) and continuous light monitoring with cumulative recording of the delivered light dose (Friauf *et al.*, submitted for publication) will further contribute to uniform and appropriate light delivery.

mTHPC-PDT following surgical tumour resection deserves further evaluation in good risk patients with diffuse malignant mesothelioma.

The authors thank P. Cotting, GMP SA, Lausanne, Switzerland, Dr T. Sonderegger, Hospital Pharmacy, Inselspital, Berne, Switzerland and Dr B. Reynolds, Scotia Pharmaceuticals Ltd, Guildford, UK for their helpful advice and assistance.

### References

BERENBAUM, M.C. (1989). Comparison of hematoporphyrin derivatives and new photosensitizers. In *Photosensitizing Compounds: Their Chemistry, Biology and Clinical Use*. Ciba Foundation Symposium 146, p. 33. John Wiley and Sons Inc: Chichester.

DELANEY, T.F., SMITH, P.D., THOMAS, G.F. & 6 others. A light diffusing device for intraoperative photodynamic therapy in the peritoneal or pleural cavity. (Submitted for publication).

DOUGHERTY, T.J., POTTER, W.R. & BELLNIER, D. (1990). Photodynamic therapy for the treatment of cancer: current status and advances. In *Photodynamic Therapy of Neoplastic Disease*, Kessel, D. (ed.) Vol.1, p. 1. CRC Press Inc.: Boston.

FABER, L.P. (1988). Surgical treatment of asbestos-related disease of the chest. *Surg. Clin. N. Am.*, **68**, 525.

FEINS, R.H., HILF, R., ROSS, H. & GIBSON, S.L. (1990). Photodynamic therapy for human malignant mesothelioma in the nude mouse. *J. Surg. Res.*, **49**, 311.

FRIAUF, W.S., SMITH, P.E., RUSSO, A. & 6 others. Light monitoring in photodynamic therapy. (Submitted for publication).

GOMER, C.J. & DOUGHERTY, T.J. (1979). Determination of [3H]- and [14C]-hematoporphyrin derivative in malignant and normal tissue. *Cancer Res.*, **39**, 149.

HERRERA-ORNELAS, L.H., PETRELLI, N.J., MITTELMAN, A., DOUGHERTY, T.J. & BOYLE, D.G. (1986). Photodynamic therapy in patients with colorectal cancer. *Cancer*, **57**, 677.

LERNER, J.H., SCHOENFELD, D.A., MARTIN, A., FALKSON, G. & BORDEN, E. (1983). Malignant mesothelioma. *Cancer*, **52**, 1981.

LOFGREN, L., LARSSON, M., THANING, L. & HALLGREN, S. (1991). Transthoracic endoscopic photodynamic treatment of malignant mesothelioma (letter). *Lancet*, **337**, 359.

MOAN, J., PENG, Q., EVENSEN, J.F., BERG, K., WESTERN, A. & RIMINGTON, C. (1987). Photosensitizing efficiencies, tumor- and cellular uptake of different photosensitizing drugs relevant for photodynamic therapy of cancer. *Photochem. Photobiol.*, **46**, 713.

NAMBISAN, R.N., KARAKOUSIS, C.P., HOLYOKE, E.D. & DOUGHERTY, T.J. (1988). Intraoperative photodynamic therapy for retroperitoneal sarcomas. *Cancer*, **61**, 1248.

NELSON, J.S., ROBERTS, W.G., LIAW, L.H. & BERNS, M.W. (1990). Cellular and tumor model studies using several PDT sensitizers. In *Photodynamic Therapy of Neoplastic Disease*, Kessel, D. (ed.) Vol. 1, p. 147. CRC Press Inc.: Boston.

PANDEY, R.K., BELLNIER, D.A., SMITH, K.M. & DOUGHERTY, T.J. (1991). Chlorin and porphyrin derivatives as potential photosensitizers in photodynamic therapy. *Photochem. Photobiol.*, **53**, 65.

PASS, H.I., TOCHNER, Z., DELANEY, T. & 4 others (1990). Intraoperative photodynamic therapy for malignant mesothelioma; letter to the editor. *Ann. Thorac. Surg.*, **50**, 687.

SINDELAR, W.F., DELANEY, T.F., TOCHNER, Z. & 6 others (1991). Technique of photodynamic therapy for disseminated intraperitoneal malignant neoplasms. *Arch. Surg.*, **126**, 318.

WILSON, B.C. & PATTERSON, M.S. (1990). The determination of light fluence distribution in photodynamic therapy. In *Photodynamic Therapy of Neoplastic Disease*, Kessel, D. (ed.) Vol. 1, p. 129. CRC Press Inc.: Boston.


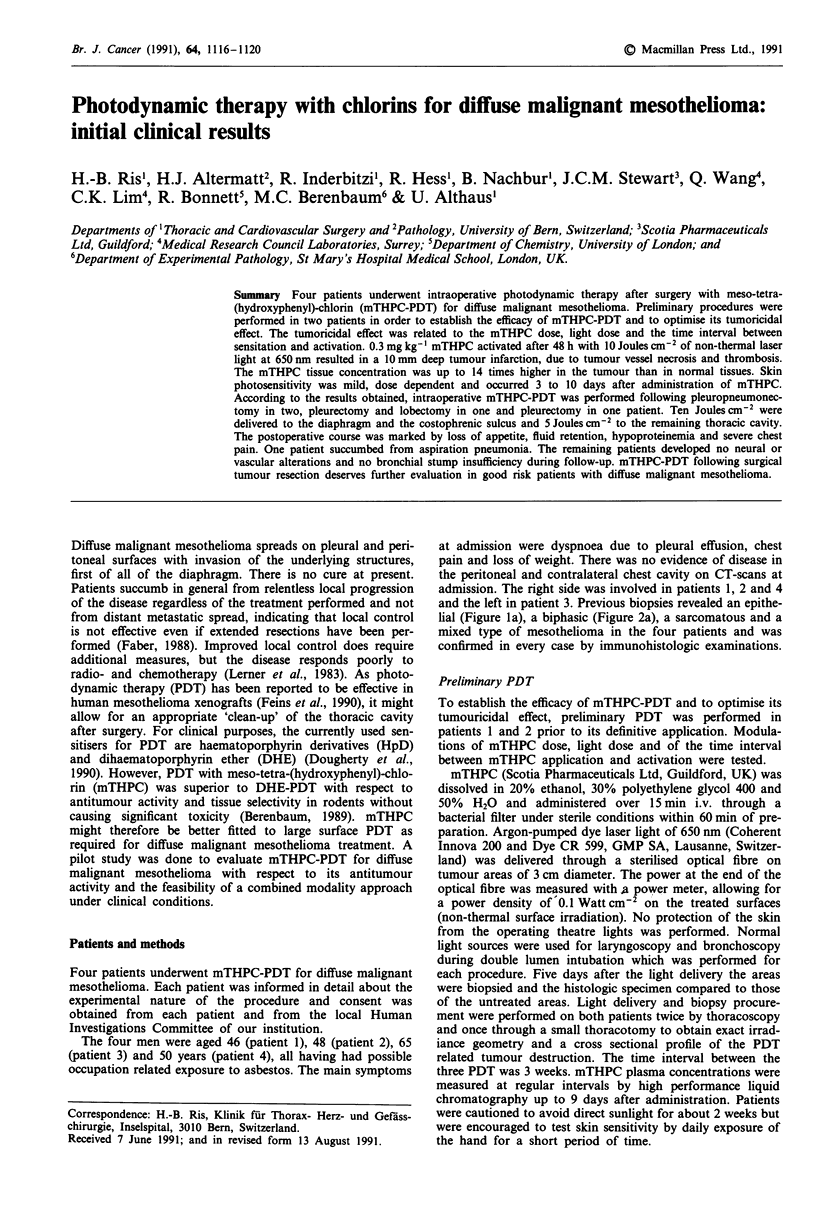

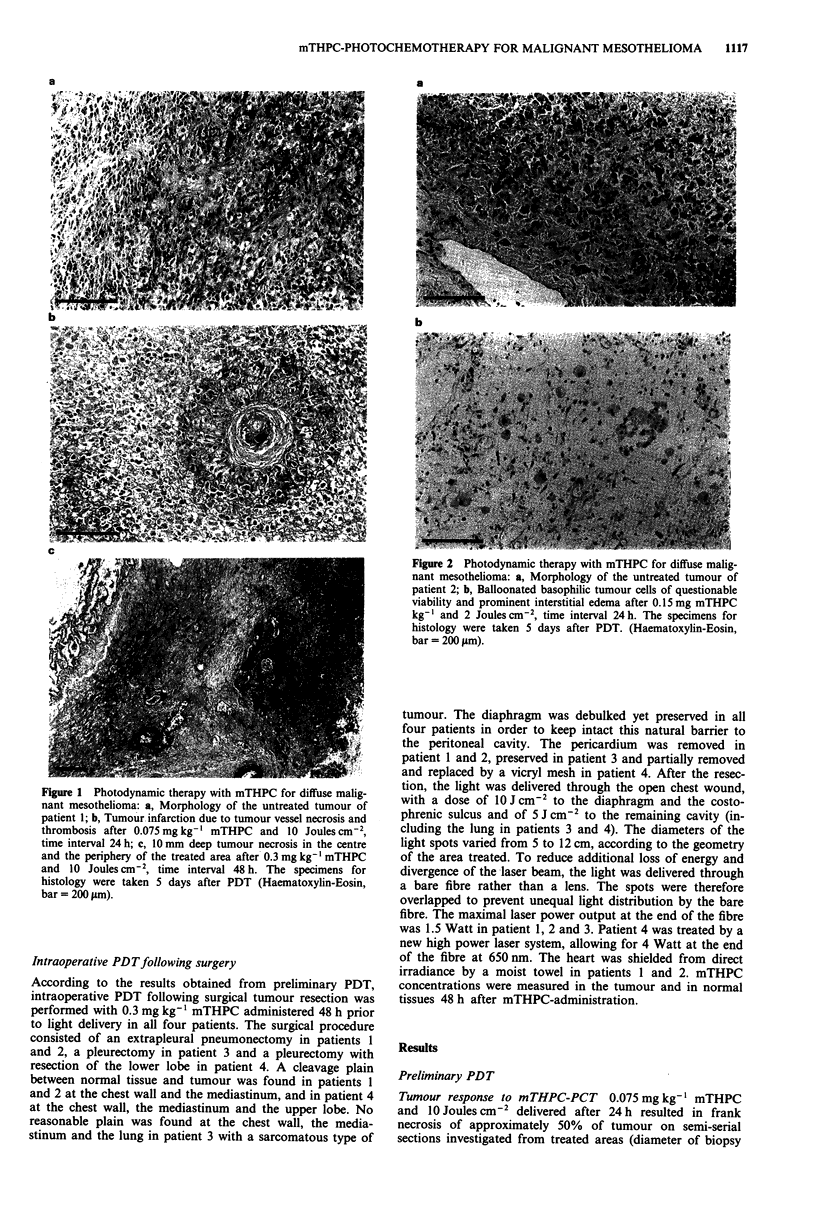

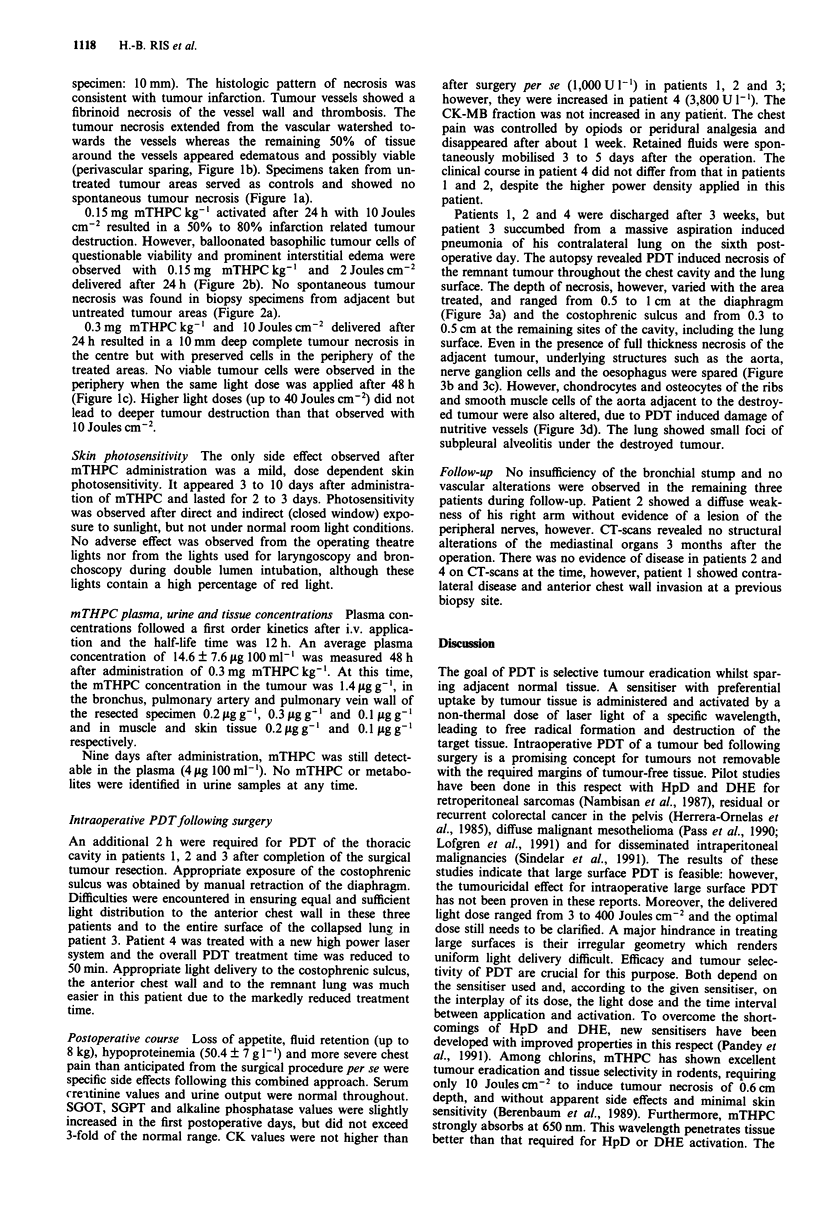

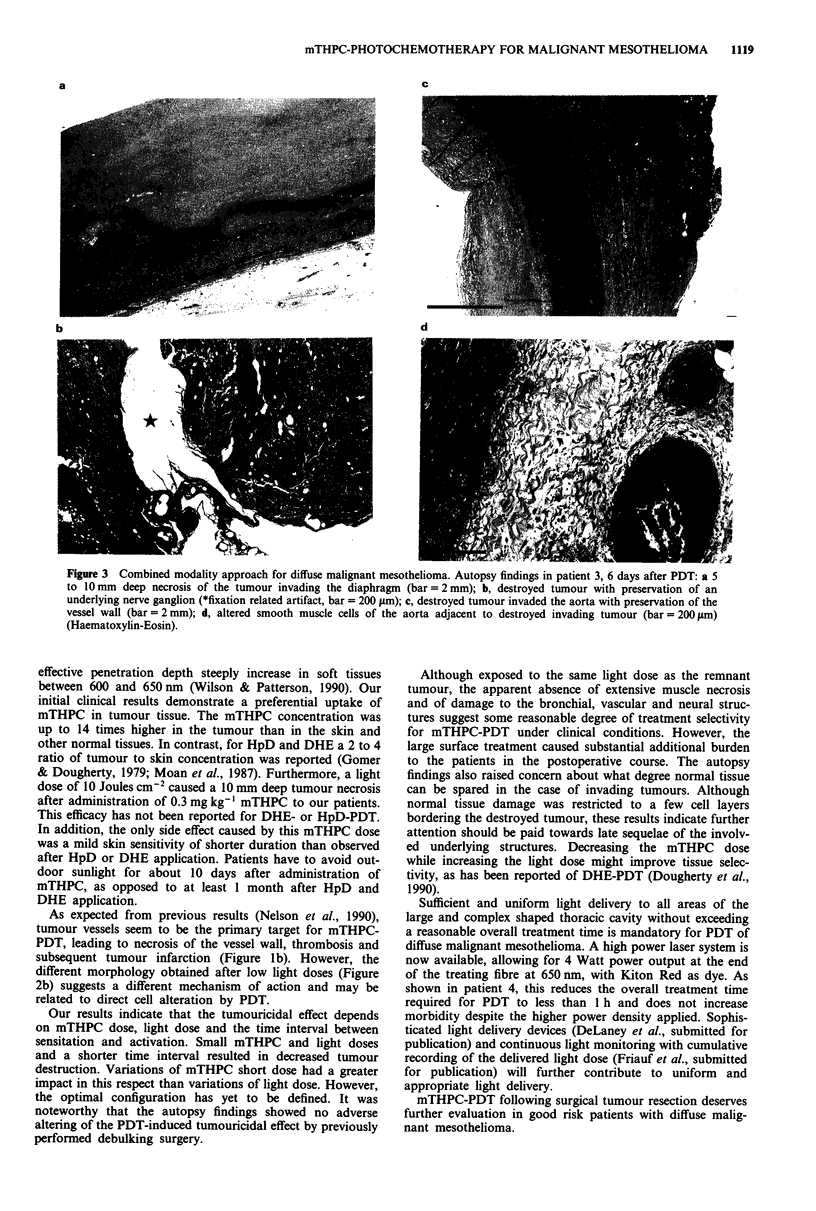

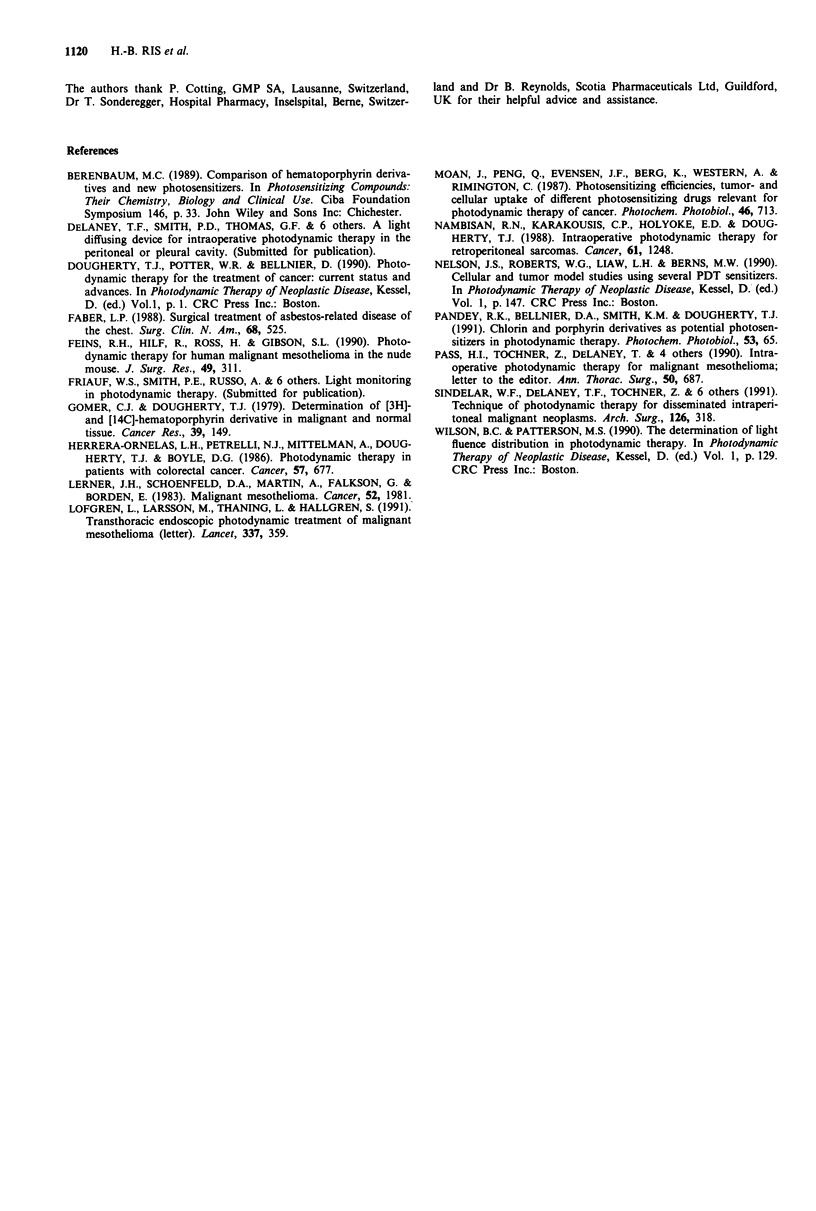


## References

[OCR_00473] Faber L. P. (1988). Surgical treatment of asbestos-related disease of the chest.. Surg Clin North Am.

[OCR_00477] Feins R. H., Hilf R., Ross H., Gibson S. L. (1990). Photodynamic therapy for human malignant mesothelioma in the nude mouse.. J Surg Res.

[OCR_00493] Herrera-Ornelas L., Petrelli N. J., Mittelman A., Dougherty T. J., Boyle D. G. (1986). Photodynamic therapy in patients with colorectal cancer.. Cancer.

[OCR_00496] Lerner H. J., Schoenfeld D. A., Martin A., Falkson G., Borden E. (1983). Malignant mesothelioma. The Eastern Cooperative Oncology Group (ECOG) experience.. Cancer.

[OCR_00499] Lofgren L., Larsson M., Thaning L., Hallgren S. (1991). Transthoracic endoscopic photodynamic treatment of malignant mesothelioma.. Lancet.

[OCR_00504] Moan J., Peng Q., Evensen J. F., Berg K., Western A., Rimington C. (1987). Photosensitizing efficiencies, tumor- and cellular uptake of different photosensitizing drugs relevant for photodynamic therapy of cancer.. Photochem Photobiol.

[OCR_00511] Nambisan R. N., Karakousis C. P., Holyoke E. D., Dougherty T. J. (1988). Intraoperative photodynamic therapy for retroperitoneal sarcomas.. Cancer.

[OCR_00520] Pandey R. K., Bellnier D. A., Smith K. M., Dougherty T. J. (1991). Chlorin and porphyrin derivatives as potential photosensitizers in photodynamic therapy.. Photochem Photobiol.

[OCR_00524] Pass H. I., Tochner Z., DeLaney T., Smith P., Friauf W., Glatstein E., Travis W. (1990). Intraoperative photodynamic therapy for malignant mesothelioma.. Ann Thorac Surg.

[OCR_00529] Sindelar W. F., DeLaney T. F., Tochner Z., Thomas G. F., Dachoswki L. J., Smith P. D., Friauf W. S., Cole J. W., Glatstein E. (1991). Technique of photodynamic therapy for disseminated intraperitoneal malignant neoplasms. Phase I study.. Arch Surg.

